# Relation between Increased IL-10 Levels and Malaria Severity: A Systematic Review and Meta-Analysis

**DOI:** 10.3390/tropicalmed8010035

**Published:** 2023-01-03

**Authors:** Phoomjai Sornsenee, Polrat Wilairatana, Kwuntida Uthaisar Kotepui, Frederick Ramirez Masangkay, Chonticha Romyasamit, Manas Kotepui

**Affiliations:** 1 Department of Family and Preventive Medicine, Faculty of Medicine, Prince of Songkla University, Songkhla 90110, Thailand; 2 Department of Clinical Tropical Medicine, Faculty of Tropical Medicine, Mahidol University, Bangkok 10400, Thailand; 3 Medical Technology, School of Allied Health Sciences, Walailak University, Tha Sala, Nakhon Si Thammarat 80160, Thailand; 4 Department of Medical Technology, University of Santo Tomas, Manila 1000, Philippines

**Keywords:** severe malaria, complicated malaria, marker, interleukin-10

## Abstract

The roles of anti-inflammatory cytokines in the pathogenesis of severe malaria have been widely studied, and the role of IL-10 in the pathogenesis of severe malaria remains unclear. Therefore, we performed a systematic review and meta-analysis to determine the difference in IL-10 levels between patients with severe malaria and those with non-severe malaria. The search for relevant studies was performed using PubMed, Scopus, and Embase from 1 February 2022 to 12 February 2022. The quality of the included studies was assessed according to the guidelines of the Strengthening the Reporting of Observational Studies in Epidemiology. The random-effects model was used to estimate the pooled effect. In all, 1215 studies were identified, and 19 were included in the quantitative syntheses. The results showed that patients with severe malaria had a higher IL-10 level than those with non-severe malaria (*p* = 0.03, pooled standardized mean difference: 0.74, 95% CI: 0.08–1.40, *I*^2^: 97.22%, 19 studies/21 sub studies). The meta-analysis results demonstrated increased IL-10 levels in patients with severe malaria compared with those with non-severe malaria. However, with the heterogeneity of the meta-analysis results, further studies are required to confirm the changes in the IL-10 levels according to the severity of malaria and to investigate whether a combination of other severity parameters with IL-10 levels could be an alternative marker for severe malaria.

## 1. Background

Malaria is the major cause of death in children younger than 5 years of age in Africa, with an estimated 241 million malaria cases and 627,000 deaths in 2020 worldwide [1]. While *Plasmodium falciparum* is the primary cause of malaria in Africa, *Plasmodium vivax* is the primary cause of malaria outside of Africa [2]. Malaria is also caused by other *Plasmodium* species, including *P. malariae* and *P. ovale*, but these cases are rare [3]. Nevertheless, mixed infections of *P. falciparum* and other less common *Plasmodium* species could lead to severe outcomes in patients [4].

Both pro- and anti-inflammatory cytokines play a vital role in the human immune response to malaria parasites [5]. The human immune response to malaria parasites depends on the person’s age, acquisition of immunity, the host, the genetics of the parasites, and the geographical location [6,7]. Although the role of inflammatory cytokines in the pathogenesis of malaria is less well defined, a recent review suggested that pro-inflammatory cytokines, such as tumor necrosis factor (TNF)-α, interferon-gamma (IFN)-γ, interleukin (IL)-6, and IL-8 play a role as proinflammatory cytokines; meanwhile, as the transforming growth factor, (TGF-β) and IL-10 play a role as anti-inflammatory cytokines in the pathophysiology of malaria [8]. Children with cerebral malaria had higher cerebrospinal fluid levels of IL-6, IL-8, granulocyte-colony stimulating factor (G-CSF), TNF-α, and the IL-1 receptor antagonist than those with noncerebral malaria [9]. However, the IL-10 levels were comparable between cerebral and non-cerebral malaria [9]. Another study showed significantly elevated IL-6 and IL-10 levels in children with severe malaria compared with uncomplicated malaria [10]. Another review suggested that IL-10 along with other immunoregulatory cytokines mediate the development of immunity against malaria, but the full impact of the production of IL-10 during malaria infection is still unclear [11]. Increased IL-10 levels were observed in children with severe or moderate anemia than in those with uncomplicated malaria [12]. Similar to the IL-10 levels, the IL-6 levels were seen to be higher in children with severe malaria cases than those with uncomplicated malaria. Nevertheless, the IFN-γ levels were significantly lower in children with severe anemia than in those with uncomplicated malaria [12]. In pregnant women, TNF and IFN-γ were associated with the pathogenesis of severe malaria [13]. An analysis of the cord blood revealed that TNF, IL-1β, and IL-5 were associated with severe malaria but IL-4, IL-6, IFN-γ, and IL-10 did not relate to severe malaria [14].

The roles of pro-inflammatory and anti-inflammatory cytokines in the pathogenesis of severe malaria have been widely studied, but the role of IL-10 in the pathogenesis of severe malaria remains unclear. In addition, most studies have provided less information from a relatively small number of participants, which precludes the collection of comprehensive data on IL-10 in the pathogenesis of the severity of malaria. Thus, we performed a systematic review and meta-analysis of the available studies that compared the IL-10 levels in patients with severe malaria and in those with non-severe malaria. These studies included a larger number of participants to provide evidence-based information as to whether the IL-10 levels could be a cytokine signature used to differentiate patients with severe malaria from those with non-severe malaria.

## 2. Methods

### 2.1. Protocols and Definition

This systematic review was performed according to the Cochrane Handbook for the Systematic Reviews of Interventions [15]. This review was reported according to the guidelines of the Preferred Reporting Items for Systematic Reviews and Meta-Analyses (PRISMA) (see PRISMA 2020 Checklist) [16]. The study protocol was registered at PROSPERO (ID: CRD42022314025).

Severe *P. falciparum* malaria is defined as the presence of the *P. falciparum* asexual parasitemia with one or more complications, including impaired consciousness, prostration, multiple convulsions, acidosis, hypoglycemia, severe malarial anemia, renal impairment, jaundice, pulmonary oedema, significant bleeding, shock, or hyperparasitemia. Severe *P. vivax* malaria is defined as for *P. falciparum* malaria but with no parasite density thresholds [17]. Severe *P. knowlesi* malaria is defined as for *P. falciparum* malaria but *P. knowlesi* hyperparasitemia is defined as a parasite density of more than 100,000/µL; jaundice with a parasite density of more than 20,000/µL [17]. Non-severe malaria is defined as the presence of *Plasmodium* asexual parasitemia without one or more of the complications listed by the WHOs criteria for severe malaria [17].

### 2.2. Eligibility Criteria

The inclusion criteria for the review were (1) studies that reported the IL-10 levels in patients with severe malaria and those with non-severe malaria, and (2) study designs that were cross-sectional, observational, cohort, or case–control studies. The exclusion criteria were studies in which (1) the data on the IL-10 levels could not be extracted, (2) no data of clinical presentation (severe or non-severe malaria) were available, (3) IL-10 was reported in pregnant women/cord blood, (4) the full text was unavailable, (5) experiments were in vitro, and (6) the IL-10 levels were measured after the treatment or intervention.

### 2.3. Information Sources

The searches were conducted using PubMed, Scopus, and Embase from 1 February 2022 to 12 February 2022. The reference lists and other sources were searched to ensure that relevant studies were not missed.

### 2.4. Search Strategy

The following combinations of search terms were used to identify potentially relevant studies: (“Interleukin 10” or IL10 or IL-10 OR “CSIF-10” OR “Cytokine Synthesis Inhibitory Factor”) AND (malaria OR Plasmodium OR “Remittent Fever” OR “Marsh Fever” OR Paludism) AND (severe OR complicated OR complication).” The search terms and their synonyms were identified by checking the Medical Subject Heading terms produced by the National Library of Medicine (Table S1).

### 2.5. Selection Process

Two authors independently selected the studies. Duplicates were first deleted from the databases. Next, the titles and abstracts of the studies were screened to find useful studies, and those that lacked pertinent information were removed. The full texts of the remaining studies were then checked, and those that did not match the inclusion criteria were eliminated with justification. Any disagreements between the authors throughout the research selection process were resolved by a consultation with the third author.

### 2.6. Data Collection Process

The data of the listed studies were gathered using a standardized Excel spreadsheet (Microsoft Corporation, Washington, DC, USA). Two authors were responsible for gathering the data independently, and another author verified the accuracy of the data.

### 2.7. Data Items

The following data were extracted: the first author’s name, year of publication, study design, study location and year of conduct, number of patients enrolled in the study, *Plasmodium* species, mean age and age group of the patients, percentage of male patients, IL-10 levels (mean ± SD or median and range in pg/mL), parasite density in severe and non-severe malaria, methods for malaria parasite detection, and methods for the IL-10’s quantification. For studies that reported the median and range of the IL-10 levels, the mean and SD were estimated from the median and the range, as described previously [18].

### 2.8. Quality of the Included Studies

The quality of the included studies was determined using the criteria of the Strengthening the Reporting of Observational Studies in Epidemiology [19]. The quality of the included studies was assessed in terms of their title and abstract, introduction, background/rationale, objectives, methods, results, discussion, and other information.

### 2.9. Effect Measures

The effect measures were the pooled mean difference in the IL-10 levels between patients with severe malaria and those with non-severe malaria.

### 2.10. Synthesis Methods

The pooled standardized mean difference (SMD, Cohen’s (d) in the IL-10 levels between patients with severe and non-severe malaria was estimated using the DerSimonian–Laird random-effects model [20]. The chi-squared (Q) test and *I*^2^ statistic were used to determine the heterogeneity of the included studies. If heterogeneity existed (Q test with a *p* of < 0.1 or *I*^2^ > 25%), meta-regression and subgroup analyses were conducted to ascertain the source(s) of the heterogeneity. The results of the individual studies and syntheses were visually displayed as a forest plot. Data synthesis was performed using Stata version 17.0 (StataCorp LLC, College Station, TX, USA).

### 2.11. Reporting Bias Assessment

The publication bias among the included studies was assessed by visualizing the funnel plot asymmetry, analyzing the small-study effect and Egger’s test, and interpreting the Contour-enhanced funnel plot.

### 2.12. Certainty Assessment

A sensitivity analysis was performed to assess the robustness of the synthesized results. A sensitivity analysis using the leave-one-out method was performed to determine the influence of each study on the pooled mean difference in the IL-10 levels.

## 3. Results

### 3.1. Search Results

**Figure 1:**
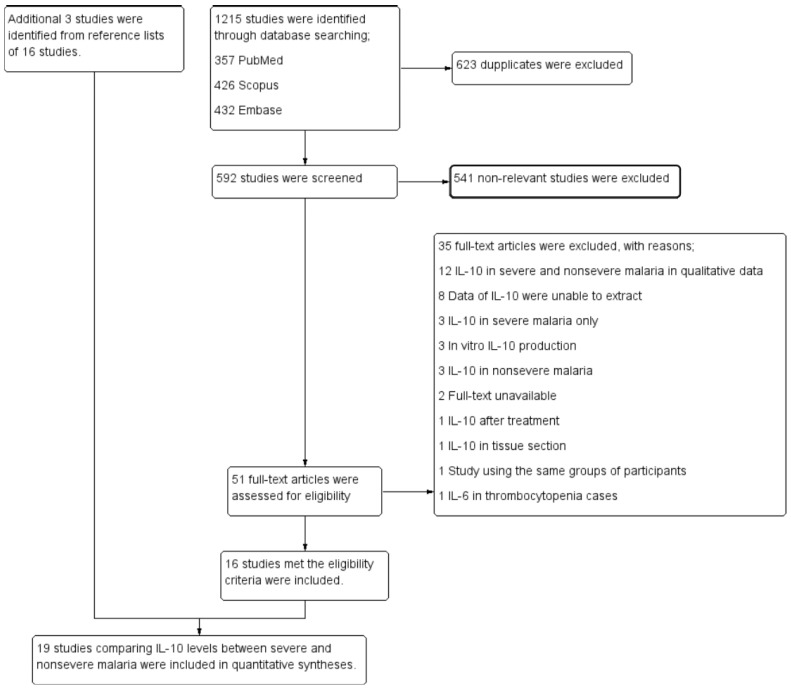
Flow diagram illustrating the selection of pertinent studies.

In all, 1215 studies were identified through database searching (357 articles from PubMed, 426 articles from Scopus, and 432 articles from Embase). Among those studies, 623 articles were duplicates and were excluded. The titles and abstracts were screened in the remaining 592 studies, after which 541 non-relevant studies were excluded. The remaining 51 articles were assessed for their eligibility by reading the full text, after which 35 articles were excluded for the following reasons: 12 studies reported IL-10 levels in severe and non-severe malaria as qualitative data, 8 studies reported IL-10 data that could not be extracted, 3 studies reported IL-10 levels in severe malaria only, 3 studies were in vitro, 3 studies reported IL-10 levels in uncomplicated malaria, the full text was unavailable in 2 studies, 1 study reported IL-10 levels after the treatment, 1 study reported IL-10 levels in tissue sections, 1 study included the same groups of participants, and 1 study reported IL-6 levels in thrombocytopenia cases. Finally, 16 studies [12,21,22,23,24,25,26,27,28,29,30,31,32,33,34,35] met the eligibility criteria and were included. An additional 3 studies [36,37,38] were identified from reference lists of 16 studies. Finally, 19 studies [12,21,22,23,24,25,26,27,28,29,30,31,32,33,34,35,36,37,38] comparing IL-10 levels between severe and non-severe malaria were included in the quantitative syntheses (Figure 1).

### 3.2. Characteristics of the Included Studies

The 19 included studies were published between 1994 and 2021. Eleven studies (57.9%) were prospective studies, six (31.6%) were retrospective studies, and two (10.5%) were cross-sectional studies. Of the included studies, 10 (52.6%) were conducted in Africa, 5 (26.3%) were conducted in Asia, 3 (15.8%) were conducted in the United States, and 1 (5.26%) was conducted in Europe. Thirteen studies (68.4%) enrolled patients infected with *P. falciparum*, four (21.1%) enrolled patients with *P. vivax*, and two (10.5%) enrolled patients with both *P. falciparum* and *P. knowlesi* [36]. Ten studies (52.6%) enrolled children, six (31.6%) enrolled adults, and three (15.8%) enrolled all age groups.

Twelve studies (63.2%) used only microscopy for the detection of malaria parasites, four (21.1%) used both microscopy and PCR, two (10.5%) used both microscopy and a rapid diagnostic test (RDT), and one study (5.26%) used three methods (microscopy/RDT/PCR) for the detection of malaria parasites. Twelve studies (63.2%) used an ELISA to quantify the IL-10 levels, whereas seven studies (36.8%) used a bead-based assay (Table 1). The details of the included studies are shown in Table S2.

### 3.3. Quality of the Included Studies

Eighteen studies (94.7%) were high-quality studies [12,21,22,23,24,25,26,27,28,29,30,31,32,34,35,36,37,38], whereas one study (5.26%) was of moderate quality [33] (Table S3).

### 3.4. Difference in IL-10 Levels between Patients with Severe and Non-Severe Malaria

**Figure 2:**
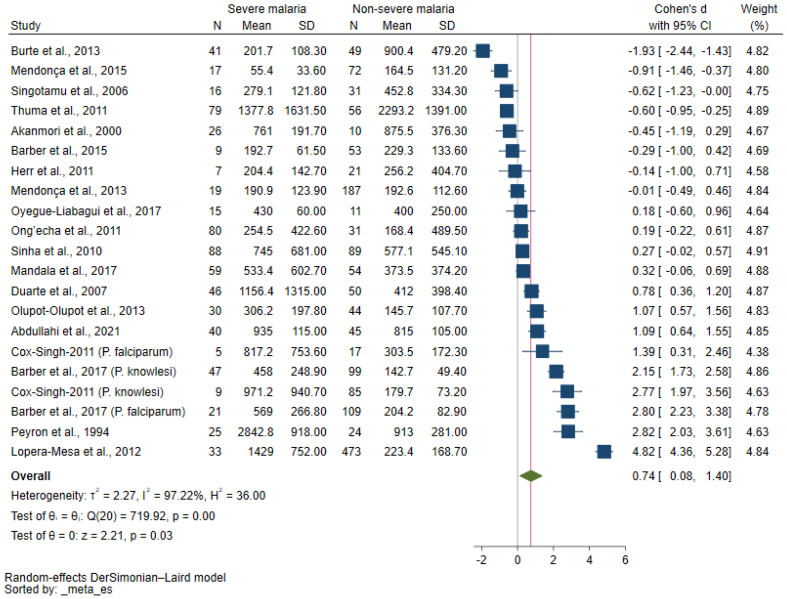
Forrest plot showing the SMDs of IL-10 levels between patients with severe and non-severe malaria. Abbreviations: CI, confidence interval; SD, standard deviation. Symbols: blue square, point estimate (SMD of IL-10); green diamond, pooled effect estimate (pooled SMD of IL-10).

The difference in the IL-10 levels between patients with severe malaria and those with non-severe malaria was estimated using data from 19 studies [12,21,22,23,24,25,26,27,28,29,30,31,32,33,34,35,36,37,38]. Two studies [24,36] that reported the IL-10 levels in both *P. falciparum* and *P. knowlesi* were analyzed separately by classification into four sub-studies. Overall, the meta-analysis results showed that patients with severe malaria had a higher SMD of the IL-10 levels than those with non-severe malaria (*p* = 0.03, pooled SMD: 0.74, 95% CI: 0.08–1.40, *I*^2^: 97.22%, 19 studies/21 sub-studies, Figure 2).

Since a high level of heterogeneity was found in the effect estimates among the included studies, meta-regression and subgroup analyses were performed to identify the source(s) of heterogeneity. The meta-regression analysis using the study designs, geographic location (continents), *Plasmodium* spp., age, mean parasitemia, and methods for the IL-10’s quantification showed that these covariates did not confound the pooled SMD (*p* > 0.05).

**Figure 3:**
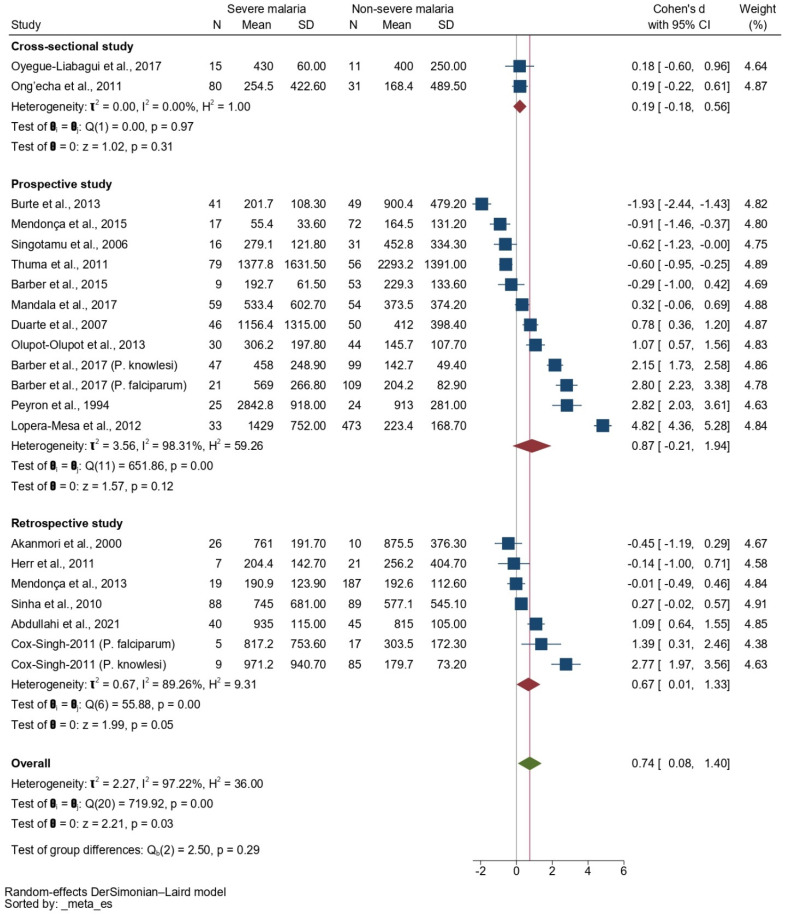
Forrest plot showing the SMDs of IL-10 levels between patients with severe malaria and non-severe malaria stratified by study design. Abbreviations: CI, confidence interval; SD, standard deviation. Symbols: blue square, point estimate (SMD of IL-10); green diamond, pooled effect estimate (pooled SMD of IL-10); red diamond, pooled effect estimate in each subgroup.

A subgroup analysis based on the study design revealed no difference in the SMD of the IL-10 levels between patients with severe and non-severe malaria in cross-sectional studies (*p* = 0.31, pooled SMD: 0.19, 95% CI: −0.1800.58, *I*^2^: 0%, 2 studies), prospective studies (*p* = 0.12, pooled SMD: 0.87, 95% CI: −0.21–1.94, *I*^2^: 98.31%, 11 studies/12 sub-studies), and retrospective studies (*p* = 0.05, SMD: 0.64, 95% CI: 0.01–1.33, *I*^2^: 89.26%, 6 studies/7 sub-studies, Figure 3).

**Figure 4:**
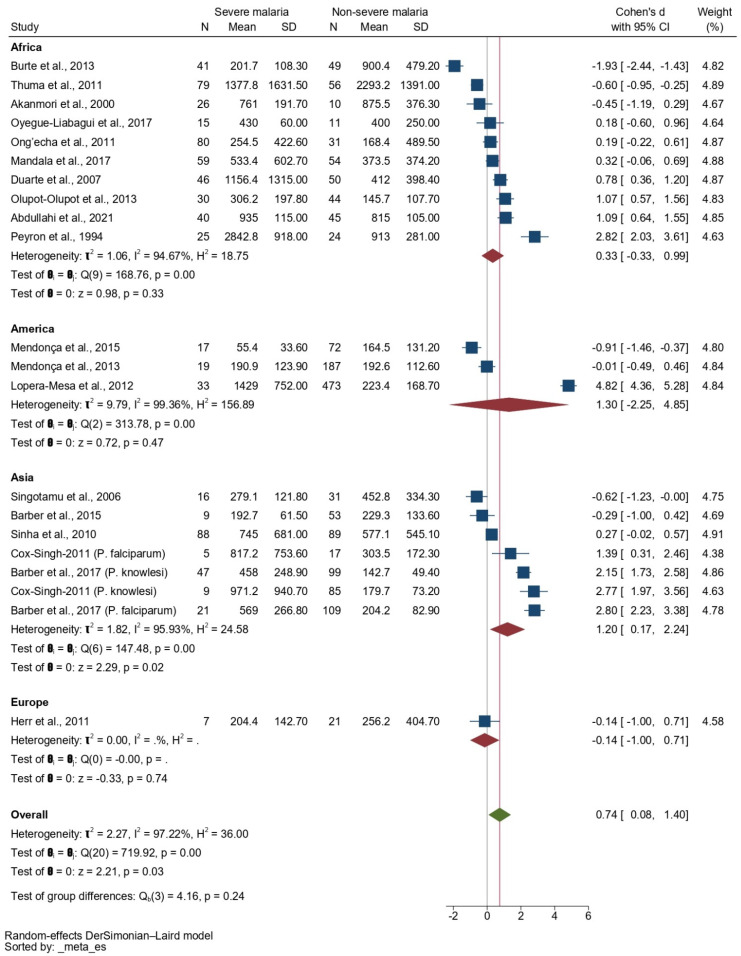
Forrest plot showing the SMDs of IL-10 levels between patients with severe malaria and patients with non-severe malaria stratified by continents. Abbreviations: CI, confidence interval; SD, standard deviation. Symbols: blue square, point estimate (SMD of IL-10); green diamond, pooled effect estimate (pooled SMD of IL-10); red diamond, pooled effect estimate in each subgroup.

The subgroup analysis based on continents revealed no difference in the SMD of the IL-0 levels between patients with severe and non-severe malaria in the studies performed in Africa (*p* = 0.33, pooled SMD: 0.33, 95% CI: −0.33–0.99, *I*^2^: 94.67%, 10 studies) and America (*p* = 0.47, pooled SMD: 1.30, 95% CI: −2.25–4.85, *I*^2^: 99.36%, 3 studies). Moreover, the meta-analysis results showed that patients with severe malaria had a higher SMD of the IL-10 level than those with non-severe malaria in the studies performed in Asia (*p* = 0.02, pooled SMD: 1.20, 95% CI: 0.17–2.24, *I*^2^: 95.93%, five studies/seven sub-studies, Figure 4).

**Figure 5:**
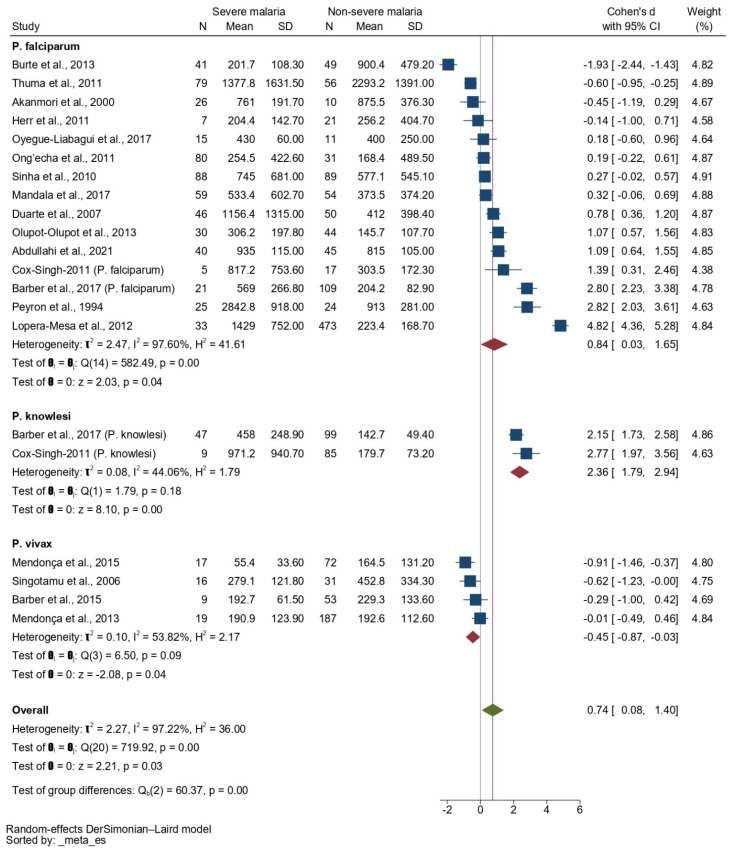
Forrest plot showing the SMDs of IL-10 levels between patients with severe malaria and those with non-severe malaria, stratified by *Plasmodium* species. Abbreviations: CI, confidence interval; SD, standard deviation. Symbols: blue square, point estimate (SMD of IL-10); green diamond, pooled effect estimate (pooled SMD of IL-10); red diamond, pooled effect estimate in each subgroup.

The subgroup analysis, based on *Plasmodium* spp. showed a higher SMD of the IL-10 level in patients with severe *P. falciparum* malaria than in those with non-severe *P. falciparum* malaria (*p* = 0.04, pooled SMD: 0.84, 95% CI: 0.03–1.65, *I*^2^: 97.60%, 15 studies). The subgroup analysis also showed a higher SMD of the IL-10 level in patients with severe *P. knowlesi* malaria than in those with non-severe *P. knowlesi* malaria (*p* < 0.001, pooled SMD: 2.36, 95% CI: 1.79–2.94, *I*^2^: 44.06%, two studies). In addition, a lower SMD of the IL-10 level was observed in patients with severe *P. vivax* malaria than in those with non-severe *P. vivax* malaria (*p* = 0.04, pooled SMD: −0.45, 95% CI: −0.87–0.03, *I*^2^: 53.82%, four studies, Figure 5).

**Figure 6:**
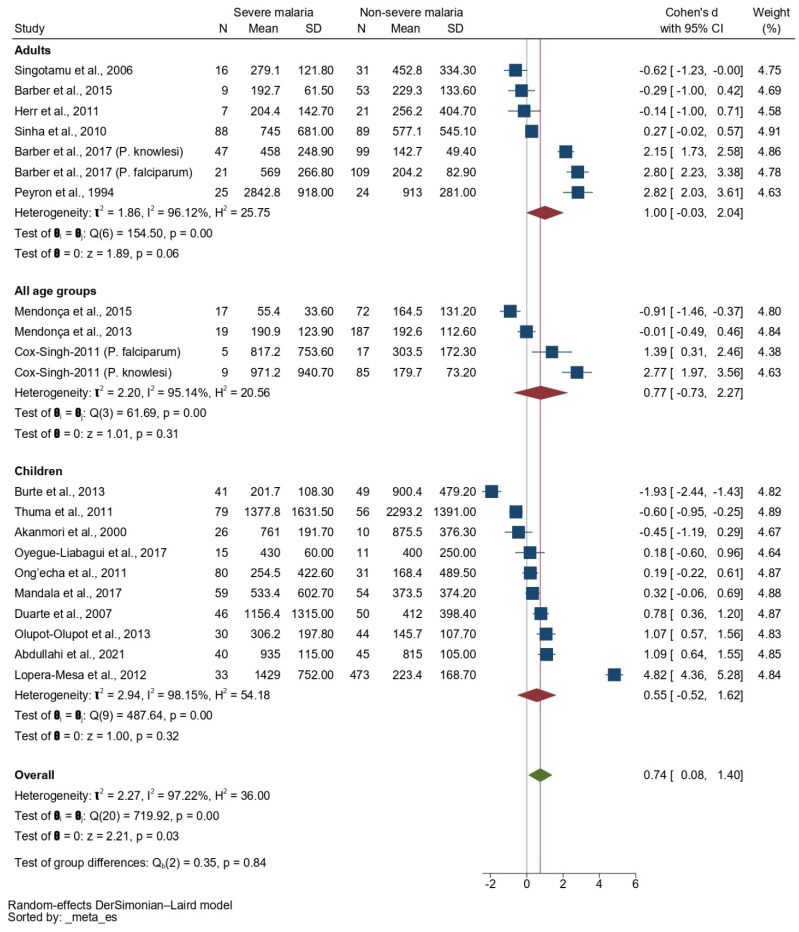
Forrest plot showing the SMDs of IL-10 levels between patients with severe malaria and those with non-severe malaria by age group. Abbreviations: CI, confidence interval; SD, standard deviation. Symbols: blue square, point estimate (SMD of IL-10); green diamond, pooled effect estimate (pooled SMD of IL-10); red diamond, pooled effect estimate in each subgroup.

A subgroup analysis based on the age groups showed no difference in the SMD of the IL-10 level between patients with severe malaria and those with non-severe malaria among studies that enrolled adults (*p* = 0.06, pooled SMD: 1.00, 95% CI: −0.03–2.04, *I*^2^: 96.12%, 7 studies), children (*p* = 0.32, pooled SMD: 0.55, 95% CI: −0.52–1.62, *I*^2^: 98.15%, 10 studies), and participants of all age groups (*p* = 0.31, pooled SMD: 0.77, 95% CI: −0.73–2.27, *I*^2^: 61.69%, 3 studies/4 sub-studies, Figure 6).

**Figure 7:**
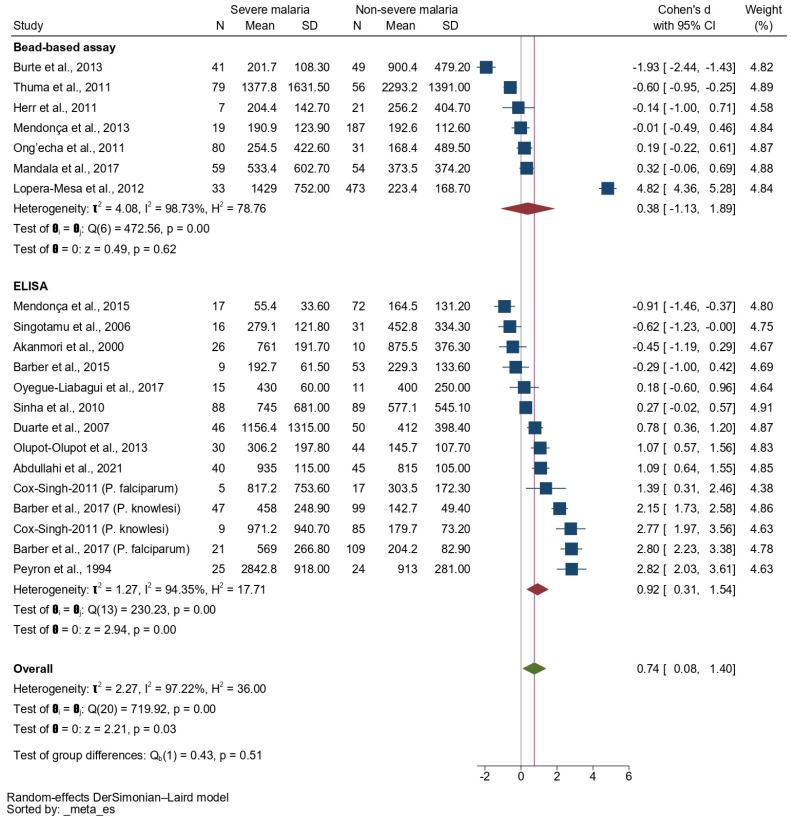
Forrest plot showing the SMDs of IL-10 levels between patients with severe malaria and patients with non-severe malaria stratified by methods used for IL-10 detection. Abbreviations: CI, confidence interval; SD, standard deviation. Symbols: blue square, point estimate (SMD of IL-10); green diamond, pooled effect estimate (pooled SMD of IL-10); red diamond, pooled effect estimate in each subgroup.

Subgroup analysis based on the methods used for the IL-10’s quantification showed a higher SMD of the IL-10 level in patients with severe malaria than in those with non-severe malaria among studies that used an ELISA for the IL-10’s quantification (*p* < 0.01, pooled SMD: 0.92, 95% CI: 0.31–1.54, *I*^2^: 94.35%, 12 studies/14 sub-studies). No difference was found in the SMD of the IL-10 level between patients with severe malaria and those with non-severe malaria among studies that used bead-based assays for the IL-10’s quantification (*p* = 0.62, pooled SMD: 0.38, 95% CI: −1.13–1.89, *I*^2^: 98.73%, seven studies, Figure 7).

### 3.5. Sensitivity Analysis

**Figure 8:**
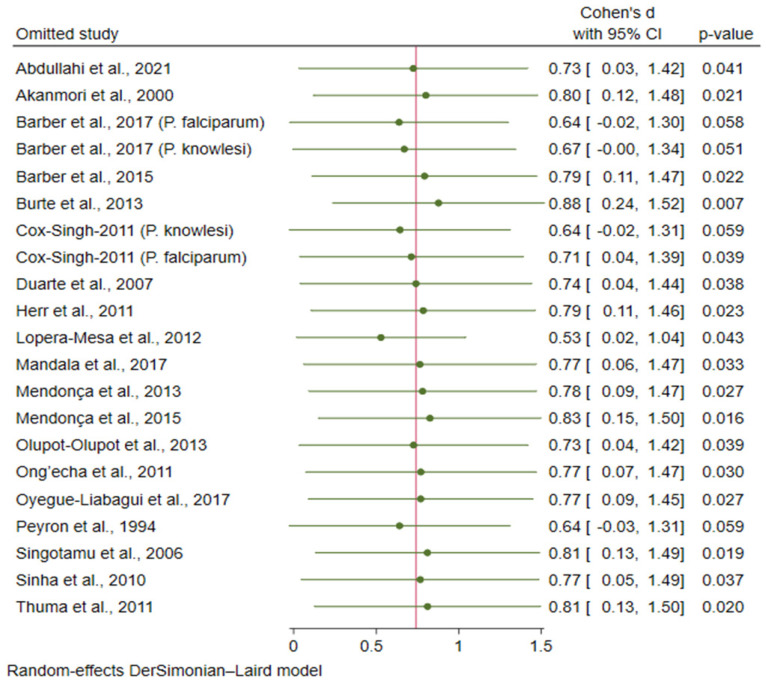
Leave-one-out analysis demonstrating the outliers in the meta-analysis. Abbreviations: CI, confidence interval. Symbols: green dot, pooled effect estimate in each re-run analysis.

The leave-one-out method was applied to test the robustness of the meta-analysis results. The results showed that when each study was omitted from the analysis, the meta-analysis results of the IL-10 levels showed outliers (*p* < 0.05 or *p* > 0.05 in re-run analyses, Figure 8).

### 3.6. Publication Bias

**Figure 9:**
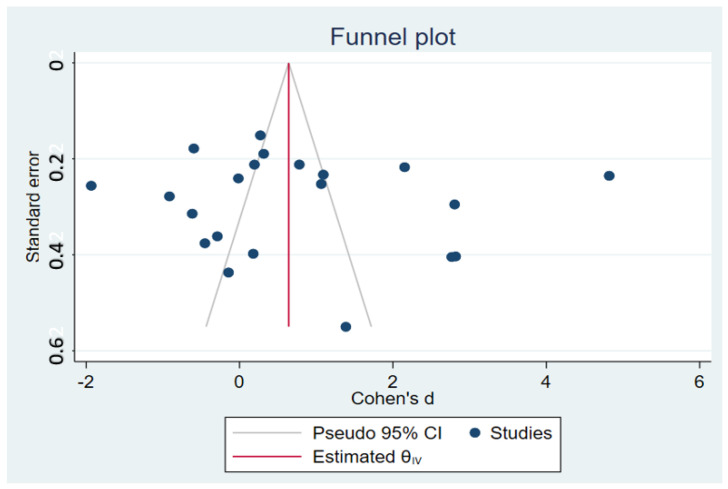
The funnel plot of each study depicts the distribution of the SMD of IL-10 levels between severe and non-severe malaria. The funnel plots show the asymmetric distribution of the SMDs of IL-10 levels and their standard error (se).

**Figure 10:**
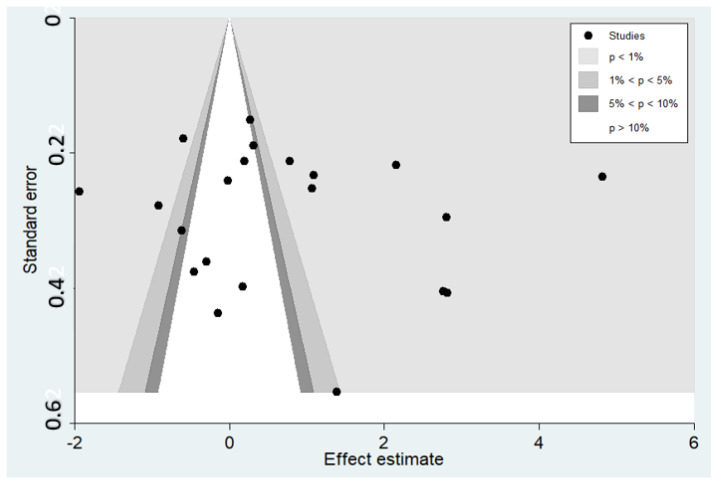
Contour-enhanced funnel plot illustrating the distribution of the SMD of IL-10 levels and their standard error (se) in significant and non-significant areas.

The funnel plot demonstrated the asymmetrical distribution of the SMDs in the IL-10 between severe and non-severe malaria from the middle line (pooled SMD) (Figure 9). The result of Egger’s test demonstrated no small-study effects (*p* = 0.653). The results of the Contour-enhanced funnel plot showed the distribution of the SMDs of the IL-10 in significant and non-significant areas, which indicates that the asymmetry of the funnel plot was likely caused by a publication bias, the heterogeneity of the SMD from the included studies, or other causes (Figure 10). After the publication bias was adjusted by the trim and fill method, the pooled SMD in the IL-10 levels between severe and non-severe malaria was 0.743 95% CI: 0.085–1.401).

## 4. Discussion

The present meta-analysis results demonstrated that patients with severe malaria had a higher mean IL-10 level than those with non-severe malaria. This suggests the possibility of using IL-10 as a severity marker for malaria. The high levels of IL-10 in patients with severe malaria indicate the imbalance of this cytokine in the pathogenesis of severe malaria [12,39]. T cell senescence together with alterations in the IL-10 might lead to a major immune dysfunction [12] and, subsequently, the development of severe malaria. IL-10 has been reported to be involved in severe malaria, particularly severe anemia. In severe anemia, low IL-10 levels might allow patients to produce enough TNF to interfere with erythropoiesis and erythrophagocytosis [22]. Furthermore, previous studies showed higher IL-10/TNF-α levels in children with severe malaria than in those with a non-severe disease [40], and these cytokines were also higher in patients who died than in those who survived [41]. Hence, alterations in the anti-inflammatory cytokine IL-10 may diminish the uncontrolled immune responses and contribute to the pathogenesis of severe malaria. In the different severity of clinical malaria, such as severe anemia and cerebral malaria, the IL-10 levels were much lower in severe anemia compared to cerebral malaria [22]. Therefore, the pathogenesis of severe malaria with different clinical complications may cause distinct forms of IL-10 alterations. Compared with healthy individuals, the IL-10 levels in patients with malaria were reported to be higher [21,22] and correlated with an increased parasite density [12,21]. Nevertheless, the IL-10 levels in patients with malaria were reported to be lower or similar in different studies [27,34,36,37,38].

A previous meta-analysis of 13 studies showed that patients with severe malaria had higher IL-6 levels than those with uncomplicated malaria [42]. Nevertheless, both IL-10 and IL-6 have been reported to be lower in severe anemia compared with uncomplicated malaria, which indicates the dual role of IL-6 and IL-10 in anti-inflammatory processes [37,41]. Another study suggested that the production of IL-10 was impaired in severe malaria compared with uncomplicated malaria [43]. Another study also suggested that the serum hepcidin levels were associated with the IL-6 and IL-10 levels in anti-inflammatory processes and determined the anemia status of patients [37]. Moreover, increases in the IL-6/IL-10 ratio have been reported to be a predictor of death in patients with malaria [41]. Increased IL-6 and IL-10 levels were observed in cerebral malaria compared with severe malaria without a cerebral involvement [41]. Another study in Malawian children found that the IL-10 levels were elevated in patients with cerebral malaria, severe anemia, and in those with uncomplicated malaria, which indicates higher-than-normal levels of this cytokine [27].

IL-10 can regulate the neutrophil function by the upregulation of the IL-1Ra and can limit the disease severity in patients with uncomplicated malaria [24]. In addition, the serum IL-10 and IL-1Ra concentrations are positively associated with the schizont rupture of *P. knowlesi* and *P. vivax*, which suggests that these anti-inflammatory cytokines play important roles in the schizont rupture and reinvasion processes [24]. Furthermore, IL-10 can promote natural killer cell activity, downregulate the TNF-α, IL-6, and IL-12 cytokine production, and inhibit the Th1 function [44,45]. Nevertheless, the effectiveness of IL-10 as an anti-inflammatory cytokine depends on the time of the production of IL-10, as production occurs within 7–8 h after the stimulation [46,47]. A recent study reported that Galectin-9 (Gal-9), a member of the galectin family of β-galactoside-binding animal lectins, could inhibit the production of pro-inflammatory cytokines, including TNF, IL-1α, and IL-6, and that it could enhance the production of IL-10, which indicates the balance between Gal-9 and IL-10 in acute malaria infection [48]. Gal-9 could enhance the differentiation of naïve T cells to Gal-9+, ThGal-9, and Tregs, which could express high levels of IL-10 mRNA [49]. Previous studies reported that serum IL-10 levels were positively associated with parasitemia in patients infected with *P. falciparum* [21], *P. knowlesi*, and *P. vivax* [24]. Nevertheless, another study reported that the IL-10 levels were lower as the disease severity increased [50]. This contradiction might signify that cytokine networks rather than a single cytokine contribute to severe malaria in different ways [46].

In the subgroup analysis disaggregated by age groups, no significant difference in the IL-10 levels was observed between patients with severe malaria and those with non-severe malaria among studies that enrolled adults, children, or both age groups. These results indicated that age was not the source of heterogeneity of the effect estimate among the included studies. The effect of age on the cytokine level has been reported in a previous study. IL-10 and other cytokines, such as IFN, IL-4, IL-6, and IL-12, were reported to increase with age, whereas IL-5 and TNF-α were shown to decrease with age [51]. These results indicated age-dependent changes in the severity of the disease, pathogenesis, and outcomes in patients with malaria. Moreover, increased IL-10 levels were reported to be an important indicator of severe malaria in older adults, particularly in patients with *P. knowlesi* malaria [36]. The effect of age and the risk of severe *P. knowlesi* malaria was previously suggested to be caused by higher parasitemia and greater exposure to mosquito vectors [36].

The meta-regression analysis showed that the parasitemia levels did not influence the pooled effect estimate, indicating that there was no relationship between the parasitemia and IL-10 levels among the studies included in the meta-analysis. The subgroup analysis of the study design showed no difference in the IL-10 levels between patients with severe and non-severe malaria in all types of study designs, indicating that the study design did influence the pooled effect estimate, and the study design was not the source of the heterogeneity of the meta-analysis outcome. The subgroup analysis of the geographic location also showed no difference in the IL-10 levels between patients with severe and non-severe malaria in the studies conducted in Africa, America, and Europe, but showed higher IL-10 levels in severe malaria than in uncomplicated malaria in the studies conducted in Asia. The difference in the IL-10 levels between patients with severe and non-severe malaria in the studies from different geographical areas might be attributed to an age group variance. It is well-explained that age was related to the acquisition of immunity against malaria. A review suggested that individuals, particularly children, would develop efficient immunity if they lived in areas where the transmission of malaria was reduced; meanwhile, children would develop less immunity if they lived in areas where the transmission of malaria was increased [52]. The subgroup analysis of *Plasmodium* spp. demonstrated that the IL-10 levels were higher in patients with severe *P. falciparum* and *P. knowlesi* infection. However, we found a paradoxical association between high IL-10 levels and a severe *P. vivax* infection. Compared to patients with non-severe *P. vivax* malaria, those with severe *P. vivax* malaria had lower IL-10 levels. This finding indicates that IL-10 may play a different regulatory role in severe *P. vivax* malaria compared to *P. falciparum* and *P. knowlesi* malaria. Therefore, the distinct regulatory role of the IL-10 levels in malaria may be linked with the age of the participants, areas with different transmission intensities, and *Plasmodium* spp.

In the subgroup analysis based on the method for the IL-10′s quantification, increased IL-10 levels were observed in patients with severe malaria than in those with uncomplicated malaria among studies that used an ELISA for the IL-10′s quantification; however, no difference in the IL-10 levels between patients with severe malaria and those with uncomplicated malaria was observed among studies that used a bead-based assay for the IL-10′s quantification. These subgroup analysis results did not confirm the efficacy of the two methods for the detection and quantification of IL-10. The use of ELISA for detection is suggested to be robust, easy to use, and suited for the measurement of a single cytokine, whereas bead-based assays are multiplex immunoassays for the detection of several cytokines in a single run. Bead-based assays are gaining popularity for the measurement of groups of cytokines and chemokines [53].

This systematic review has limitations in evidence and in the review processes. First, some full texts of the relevant studies that reported IL-10 in patients with severe malaria may have been missed due to the limitation of access to some specific databases. Second, a limited number of studies were included in the meta-analysis, particularly studies that investigated the IL-10 levels in patients with *P. knowlesi* and *P. vivax* malaria; thus, the pooled analysis of evidence was limited. Third, the heterogeneity of the pooled SMD among the included studies could not be sufficiently explored due to the limitation of information about the included studies. Fourth, the difference in the IL-10 levels between patients with different severe complications of malaria could not be analyzed due to the incomplete and limited information from the included studies. Fifth, the meta-analysis results may have been affected by a publication bias. Although the overall meta-analysis results indicated that IL-10 can be a potential marker for severe malaria, the sensitivity analysis demonstrated outliers that affect the robustness of the results of the meta-analysis. More information from additional studies is required to demonstrate the performance of IL-10 as a marker for severe *P. falciparum*, *P. knowlesi*, and *P. vivax* malaria. In addition, if another novel or well-studied severity parameter is considered along with the IL-10 levels, it could have more impact on the robustness of the inclusion criteria.

## 5. Conclusions

The meta-analysis results demonstrated that patients with severe malaria have increased IL-10 levels compared to those with non-severe malaria. However, with the heterogeneity of the meta-analysis results, further studies are required to confirm the changes in the IL-10 levels according to the severity of malaria and to investigate whether a combination of other severity parameters with IL-10 levels could be an alternative marker for severe malaria.

## Figures and Tables

**Table 1:** Characteristics of the included studies.

Characteristics	*n*.	%
Study designs		
Prospective study	11	57.9
Retrospective study	6	31.6
Cross-sectional studies	2	10.5
Study areas		
Africa	10	52.6
Asia	5	26.3
America	3	15.8
Europe	1	5.26
*Plasmodium* spp.		
*P*. *falciparum*	13	68.4
*P*. *vivax*	4	21.1
*P*. *falciparum* and *P*. *knowlesi*	2	10.5
Participants		
Children	10	52.6
Adults	6	31.6
All age groups	3	15.8
Methods for malaria detection		
Microscopy	12	63.2
Microscopy and PCR	4	21.1
Microscopy and RDT	2	10.5
Microscopy/RDT/PCR	1	5.26
Methods for IL-10 quantification		
ELISA	12	63.2
Bead-based assay	7	36.8

Abbreviations: ELISA, enzyme-linked immunosorbent assay; PCR, polymerase chain reaction; RDTs, rapid diagnostic tests.

## Data Availability

All data relating to the present study are available in this manuscript.

## Supplementary Materials

The following supporting information can be downloaded at: https://www.mdpi.com/article/10.3390/tropicalmed8010035/s1, Table S1. Search terms. Table S2. Details of the included studies. Table S3. Quality assessment of the included studies.

## Abbreviations

IFN: interferon gamma; IL, interleukin; IL-1Ra, interleukin-1 receptor antagonist; PRISMA, Preferred Reporting Items for Systematic Reviews and Meta-Analyses; TGF, transforming growth factor-beta; TNF-α, tumor necrosis factor alpha.
