# Supplementary material for: Relation between Increased IL-10 Levels and Malaria Severity: A Systematic Review and Meta-Analysis
Source: Trop Med Infect Dis. 2023 Jan 3;8(1):35. doi: 10.3390/tropicalmed8010035 (PMC9865813; doi:10.3390/tropicalmed8010035)
Supplement: Supplementary file 1 [file tropicalmed-08-00035-s001.zip › Table S1. Search terms.pdf]

# Relation between Increased IL-10 Levels and Malaria Severity: A Systematic Review and Meta-Analysis

**Phoomjai Sornsenee [1], Polrat Wilairatana [2], Kwuntida Uthaisar Kotepui [3], Frederick Ramirez Masangkay [4], Chonticha Romyasamit [3], and Manas Kotepui [3,*]**

[1] Department of Family and Preventive Medicine, Faculty of Medicine, Prince of Songkla University, Songkhla 90110, Thailand
[2] Department of Clinical Tropical Medicine, Faculty of Tropical Medicine, Mahidol University, Bangkok 10400, Thailand
[3] Medical Technology, School of Allied Health Sciences, Walailak University, Tha Sala, Nakhon Si Thammarat 80160, Thailand
[4] Department of Medical Technology, University of Santo Tomas, Manila 1000, Philippines
* Correspondence: manas.ko@wu.ac.th or manaskote@gmailcom

**Table S1. Search term**

| **Databases** | **Search terms/Search strategy** | **Date** |
|---|---|---|
| MEDLINE (via PubMed) | ("Interleukin 10" OR IL10 OR IL-10 OR "CSIF-10" OR "Cytokine Synthesis Inhibitory Factor") AND (malaria OR Plasmodium OR "Remittent Fever" OR "Marsh Fever" OR Paludism) AND (severe OR complicated OR complication)<br><br>Search results: 357 | 1 to 12 February 2022 |
| Scopus | ("Interleukin 10" OR IL10 OR IL-10 OR "CSIF-10" OR "Cytokine Synthesis Inhibitory Factor") AND (malaria OR Plasmodium OR "Remittent Fever" OR "Marsh Fever" OR Paludism) AND (severe OR complicated OR complication)<br><br>Search option: Title, abstract, keywords<br><br>Search results: 426 | 1 to 12 February 2022 |
| Embase | ("Interleukin 10" OR IL10 OR IL-10 OR "CSIF-10" OR "Cytokine Synthesis Inhibitory Factor") AND (malaria OR Plasmodium OR "Remittent Fever" OR "Marsh | 1 to 12 February 2022 |

| | | |
|---|---|---|
| | Fever" OR Paludism) AND (severe OR complicated OR complication)<br><br>Search results: 432 | |
